# Comparing the Efficacy and Efficiency of Human and Generative AI: Qualitative Thematic Analyses

**DOI:** 10.2196/54482

**Published:** 2024-08-02

**Authors:** Maximo R Prescott, Samantha Yeager, Lillian Ham, Carlos D Rivera Saldana, Vanessa Serrano, Joey Narez, Dafna Paltin, Jorge Delgado, David J Moore, Jessica Montoya

**Affiliations:** 1 HIV Neurobehavioral Research Program University of California, San Diego San Diego, CA United States; 2 San Diego State University/University of California San Diego Joint Doctoral Program in Clinical Psychology San Diego, CA United States; 3 Department of Medicine University of California, San Diego San Diego, CA United States; 4 Department of Psychiatry University of California, San Diego La Jolla, CA United States

**Keywords:** GenAI, generative artificial intelligence, ChatGPT, Bard, qualitative research, thematic analysis, digital health

## Abstract

**Background:**

Qualitative methods are incredibly beneficial to the dissemination and implementation of new digital health interventions; however, these methods can be time intensive and slow down dissemination when timely knowledge from the data sources is needed in ever-changing health systems. Recent advancements in generative artificial intelligence (GenAI) and their underlying large language models (LLMs) may provide a promising opportunity to expedite the qualitative analysis of textual data, but their efficacy and reliability remain unknown.

**Objective:**

The primary objectives of our study were to evaluate the consistency in themes, reliability of coding, and time needed for inductive and deductive thematic analyses between GenAI (ie, ChatGPT and Bard) and human coders.

**Methods:**

The qualitative data for this study consisted of 40 brief SMS text message reminder prompts used in a digital health intervention for promoting antiretroviral medication adherence among people with HIV who use methamphetamine. Inductive and deductive thematic analyses of these SMS text messages were conducted by 2 independent teams of human coders. An independent human analyst conducted analyses following both approaches using ChatGPT and Bard. The consistency in themes (or the extent to which the themes were the same) and reliability (or agreement in coding of themes) between methods were compared.

**Results:**

The themes generated by GenAI (both ChatGPT and Bard) were consistent with 71% (5/7) of the themes identified by human analysts following inductive thematic analysis. The consistency in themes was lower between humans and GenAI following a deductive thematic analysis procedure (ChatGPT: 6/12, 50%; Bard: 7/12, 58%). The percentage agreement (or intercoder reliability) for these congruent themes between human coders and GenAI ranged from fair to moderate (ChatGPT, inductive: 31/66, 47%; ChatGPT, deductive: 22/59, 37%; Bard, inductive: 20/54, 37%; Bard, deductive: 21/58, 36%). In general, ChatGPT and Bard performed similarly to each other across both types of qualitative analyses in terms of consistency of themes (inductive: 6/6, 100%; deductive: 5/6, 83%) and reliability of coding (inductive: 23/62, 37%; deductive: 22/47, 47%). On average, GenAI required significantly less overall time than human coders when conducting qualitative analysis (20, SD 3.5 min vs 567, SD 106.5 min).

**Conclusions:**

The promising consistency in the themes generated by human coders and GenAI suggests that these technologies hold promise in reducing the resource intensiveness of qualitative thematic analysis; however, the relatively lower reliability in coding between them suggests that hybrid approaches are necessary. Human coders appeared to be better than GenAI at identifying nuanced and interpretative themes. Future studies should consider how these powerful technologies can be best used in collaboration with human coders to improve the efficiency of qualitative research in hybrid approaches while also mitigating potential ethical risks that they may pose.

## Introduction

### Background

Qualitative methods are pivotal for the development and implementation of digital health interventions. In implementation science, qualitative methods are often used to inform, refine, and improve digital health interventions [1]. Thematic analysis can be applied to qualitative data generated from various methods or sources (eg, key informant interviews and focus groups). This flexible and broad method involves identifying, extracting, and interpreting common themes (ie, codes) within the data that are not subscribed to a particular theory [2,3]. These themes may be identified via inductive (“bottom-up”) or deductive (“top-down”) methods [2]. In the former, themes are data driven, reflecting a rich description of the overall data. In contrast, the latter is driven by existing literature and previously published health behavior models, resulting in a detailed analysis of specific data that fit within a priori coding frames.

Compared to quantitative methods, qualitative methods are often more resource and cost intensive, conflicting with the need for timely feedback in rapidly changing real-world settings (eg, changes in health care policies and patient needs). Such delays in research on evidence-based practices unfortunately minimize their relevance and applicability [4]. An emerging alternative to traditional qualitative methods includes rapid qualitative analyses, which most commonly aim to reduce the time invested in data collection, management, analysis, and interpretation [5,6]. Studies comparing rapid qualitative analyses to traditional methods have shown a good overlap between themes [1,5,7], with additional benefits such as greater data collection and decreased costs [6]. Nonetheless, ongoing challenges to rapid analyses include reduced scientific rigor (ie, trustworthiness) [6,8] and an intensified workload due to a truncated timeline [1,5].

ChatGPT (Open AI) and Google Bard (subsequently rebranded as Gemini) are 2 popular generative artificial intelligence (GenAI)–based systems that provide an interface for humans to collaborate with powerful large language models (LLMs): OpenAI’s GPT-3.5 neural engine [9] and Google’s PaLM 2 [10]; these models are trained to predict and generate humanlike textual responses by leveraging deep learning techniques on massive amounts of pre-existing textual data [11,12]. Recently, LLMs have outperformed previously developed artificial intelligence (AI) systems across different tasks spanning a wide range of disciplines [13]. There is a growing interest in exploring clinical uses for GenAI including new drug design [14] and brain tumor imaging [15]. In the digital health setting, GenAI apps offer individualized information to users on diverse health topics including chronic and infectious diseases or healthy lifestyle choices [16]. In research, GenAI functions can range from summarizing literature and analyzing data (including coding) to identifying research gaps and drafting papers [17]. Despite these powerful uses, questions remain about the reliability of GenAI as a research tool, given the possibility that GenAI generates incorrect text (eg, “hallucinations”) and distorts scientific facts [17].

In the realm of qualitative research, the interpretation of observed events introduces significant subjectivity. Triangulation [8,18] is a strategy to improve the validity or efficacy of qualitative analysis by integrating information from different sources (eg, human- vs computer-derived codebooks), thereby leveraging the advantages of multiple data analysis methods. For example, Firmin et al [19] found that human-generated thematic codes and software-driven categories were highly correlated for concrete constructs but highlighted unique subjective or abstract constructs. Qualitative analysis by human coders targets meanings and interpretations, whereas LLMs target structural and logical elements of language [11]. To date, there have only been 2 known studies that have recently demonstrated and evaluated the efficacy of applying GenAI for qualitative research compared to human analysts. de Paoli [11] explored whether the LLM underlying ChatGPT could be used to conduct inductive thematic analysis. The results suggested that at least some of the themes previously identified by human analysts in the contexts of education and psychology were able to be reproduced by GenAI and warranted further exploration and methodological considerations [11]. Alternatively, Hamilton et al [20] leveraged ChatGPT to conduct a phenomenological qualitative analysis of significant statements from the interview transcripts nested within a guaranteed income program evaluation and compared its identified themes to those generated by human analysts. They similarly found promising similarities in identified themes as well as discrepancies such as limited contextual understanding from ChatGPT.

Although substantial debate remains as to how to best evaluate the methodological rigor or trustworthiness of qualitative research, the accuracy in which findings reflect the data (ie, efficacy) and the reliability within analytic procedures are prominent considerations [21]. The extent to which GenAI can produce rigorous and trustworthy qualitative research while reducing the time and resource burden of current qualitative methods remains open to exploration, particularly within the context of health-related research.

### Objectives

The primary objectives of our study were to assess the consistency and reliability of thematic analysis conducted by ChatGPT, Bard, and human coders following both inductive and deductive approaches. In this paper, we have described and compared the methods that we used among humans and GenAI to contribute to the growing body of literature on the wide-ranging applications of GenAI for qualitative analysis in digital health research. Specifically, we aimed to compare both the consistency in identifying broad themes essential to qualitative research and the reliability in coding between methods (ie, humans, ChatGPT, and Bard). Furthermore, we additionally examined the difference in human resources required (ie, time spent on the analysis) between the methods for both approaches.

## Methods

### Qualitative Data

The qualitative data for this study consisted of 40 short (<160 characters; 5-14 words in length) SMS text message prompts used in a previous study evaluating an SMS text messaging intervention (individualized texting for adherence building; iTAB) to promote antiretroviral medication adherence among people with HIV who use methamphetamine [22]. The iTAB messages draw from various health behavior models including the health belief model [23], theory of planned behavior [24], social cognitive theory [25], and attitude–social influence–efficacy model [26]. During the development of iTAB, sample messages were tested among people with HIV who provided feedback; participant feedback was subsequently used to adapt the SMS text messages. These SMS text messages served as the foundation for the final, streamlined version [27]. For this study, the 40 short SMS text messages were the qualitative data being analyzed. We used SMS text message prompts as opposed to participant-generated qualitative responses in our analyses, as GenAI services record all data entered to further train LLMs.

### Ethical Considerations

Presently, the use of participant-generated SMS text messages would violate the protection of confidentiality agreements as the consent forms approved by our institutional review board did not specify that participant-generated qualitative data would be uploaded to third-party vendors. We believe that using the 40 short SMS text message prompts provides a proxy for qualitative data to model how GenAI and LLMs compare in detecting shared themes among the SMS text messages compared to human-conducted thematic analysis. This was an institutional review board–exempt study as there were no data from human participants involved.

### GenAI Services

#### Overview

We used 2 commercially available GenAI services: ChatGPT-3.5 (OpenAI) and Bard (Google). ChatGPT-3.5 leverages OpenAI’s proprietary LLM (GPT-3.5), which was trained using reinforcement learning from human feedback [28], a method that provides rewards to reinforce learning. Bard is powered by Google’s proprietary LLM (PaLM 2) [10], a transformer-based model that enables it to conduct advanced reasoning tasks including classification and language generation. Both LLMs are currently free and open to the public.

#### GenAI: Inductive Thematic Analysis Procedures

**Figure 1:**
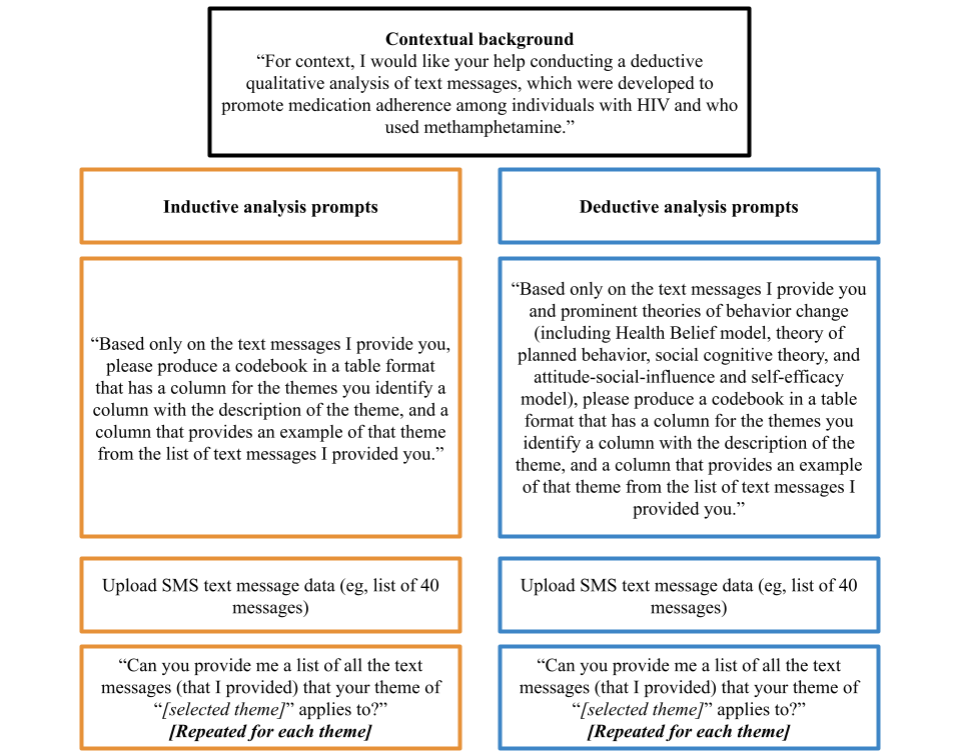
Generative artificial intelligence thematic analysis instruction prompts.

ChatGPT and Bard were given identical prompts to conduct inductive thematic analysis. Before providing the SMS text message prompts, both GenAI services were prompted with contextual information on the study and a description of the procedures, which the independent human analyst asked GenAI to perform (Figure 1). Following this contextual information and instructions, the SMS text messages were copied into the GenAI interface all at once (ie, as a list of 40 messages), and the model exported the requested 3-column codebook table. We then provided additional instructions to have the GenAI label the SMS text messages based on shared themes (Figure 1).

#### GenAI: Deductive Thematic Analysis Procedures

ChatGPT and Bard were given identical prompts to conduct deductive thematic analysis. Both GenAI services were prompted with the same background contextual information and instructions as the inductive thematic analysis prompts. All SMS text messages for analyses were similarly copied all at once into the GenAI. However, the deductive approach additionally requested that SMS text messages be categorized using constructs from relevant theories of behavior change (such as medication adherence) including the health belief model [23], theory of planned behavior [24], social cognitive theory [25], and attitude–social influence–efficacy model) [26] (Figure 1).

#### Training of Human Coders and Analyst

In terms of training, all the 4 human coders responsible for the qualitative analysis had been previously trained by the senior author on the proper conduct of qualitative analysis on prior studies, as well as had attended formal external webinars on qualitative coding and analysis. Each thematic analysis was conducted by 2 research team members consisting of a clinical psychology doctoral student and a research assistant with a bachelor’s degree. These research team members were not involved in the development or evaluation of the SMS text messaging intervention. The separate inductive and deductive human teams were thus intentionally balanced in their experience and expertise with qualitative analysis and were instructed not to discuss or collaborate on their analyses to maintain the independence of each analytic approach.

Similar to human coders, the human analyst responsible for developing the GenAI prompts used in this study had previous training and experience with qualitative analysis. The human analyst also had several years of experience working in technology development, applying emerging technologies to digital health, and incorporating general guidelines on prompt engineering in the context of health care [29]. Specifically, the analyst incorporated guidelines such as being specific, providing the setting and context, identifying the overall goal first, and requesting examples to inform their prompts.

#### Procedures for Human Inductive Thematic Analysis

For inductive thematic analysis, both members were given contextual background on the SMS text messaging study and were instructed to independently develop their own codebook. Once each member developed an initial codebook, they were instructed to come to a consensus on a final codebook. Following agreement on a final codebook, each team member applied the codebook to group the SMS text messages and began to search for themes. Finally, both team members compared their application of the final codebook, resolved disagreements in coding, and ultimately came to a consensus on the broader themes derived by summarizing and collapsing codes. This process was consistent with the basic steps of a thematic analysis [2].

#### Procedures for Human Deductive Thematic Analysis

For deductive thematic analysis, both research members were given the same contextual study information as the inductive thematic analysis team. The deductive analysis team was then given a list of a priori codes based on the theories of behavior change including health belief model [23], theory of planned behavior [24], social cognitive theory [25], and attitude–social influence–efficacy model [26]. Next, the deductive analysis team was instructed to independently develop a codebook considering the key constructs of the theories of behavior change and was suggested a priori codes. Once both team members independently developed a codebook, they were instructed to compare codebooks and reached a consensus on a final codebook. Following agreement on a final codebook, each team member applied the codebook to group the text messages and began searching for themes. Both team members then compared their application of the final codebook, resolved any discrepancies in coding, and came to a consensus on broader themes by collapsing and summarizing their codes. This process was consistent with the basic steps of a thematic analysis [2].

### Consistency and Intercoder Reliability

Consistency was defined as the extent to which the thematic findings were the same across the 2 analytic methods, as has previously been used when comparing qualitative methods [1]. We operationalized this as the percentage of themes that was shared between methods (eg, 100% consistency would suggest that the themes between the methods were identical). For example, if method A (reference method) were to identify 10 total themes and method B identified 5 of those themes, then the theme consistency would be 50% (5/10).

Intercoder reliability (ICR) was operationalized as the number of agreements in coding divided by the sum of agreements and disagreements in coding [30]; thus, a higher score equates to a greater agreement between coders. After reaching a final consensus on the codebook, an ICR was calculated. Human coders then met to discuss disagreements in the coding of the data. To compare the reliability of coding between humans and GenAI for themes that were shared by both methods, ICRs were calculated to determine the reliability between (1) human coders and Bard, (2) human coders and ChatGPT, and (3) ChatGPT and Bard. ICRs were averaged across all common themes to compute an overall ICR percentage. To qualitatively describe the extent of agreement between human and GenAI teams, the following cutoffs were used to interpret the ICRs: slight (0%-20%), fair (21%-40%), moderate (41%-60%), substantial (61%-80%), and almost perfect (81%-100%) [31]. The consistency and ICR between methods were descriptively reported and compared.

### Total Time Spent on the Analyses

After all thematic analyses had been completed, each human coder was asked to retrospectively estimate the amount of total time spent on their qualitative analyses. For the human coders, this total time included the sum of the time spent by each individual coder on their initial coding, codebook development, application of codebook, and meetings to reach a consensus on disagreements in coding and themes. The total time spent on thematic analyses using GenAI was the sum of the time taken to input the prompts, waiting for responses to generate, and compiling those responses in a spreadsheet table. The differences in the total time spent on analysis between methods were also descriptively reported and compared.

## Results

### Consistency of Inductive Thematic Analyses

In the inductive arm of our study, 7 themes were identified by the human coders following an inductive thematic analysis of the iTAB SMS text messages. These themes included “time,” “adherence,” “religious,” “community care,” “health reminder,” “warning,” and “encouragement.” Of these 7 themes identified by human coders, 5 (71%) were also consistent with the themes derived by both ChatGPT and Bard. Multimedia Appendix 1 presents a complete mapping of the inductive thematic analysis codebooks (including theme, description, and example text messages) generated by human coders, ChatGPT, and Bard.

ChatGPT’s inductive thematic analysis of the same SMS text messages identified 10 themes, which included “spirituality/higher power,” “supportive community,” “love and support from others,” “health benefits,” “reminder,” “resistance and risk to others,” “consequences of nonadherence,” “positive reinforcement,” “fun and enjoyment,” and “accountability.” Of these 10 themes, almost all (n=9, 90%) were consistent with the themes identified by our human coders. Bard’s inductive thematic analysis identified 6 themes from the SMS text messages, which included “religious/spiritual beliefs,” “social support,” “importance of taking medication,” “consequences of not taking medication,” “enjoyment,” and “personal responsibility.” Of these 6 themes, the majority (n=5, 83%) were consistent with the themes identified by human coders, and there was perfect consistency (6/6, 100%) between the themes identified by ChatGPT and Bard.

The 1 theme that ChatGPT identified that human coders did not was “accountability,” which was defined as “Messages emphasizing personal responsibility for adherence.” Bard similarly identified this theme as “personal responsibility,” which was defined as “The messages emphasize that it is important for people to take care of themselves and take their medication on their own. They also suggest that people should be proud of themselves for being adherent to their medication regimen.” The example SMS text messages provided by ChatGPT and Bard that are representative of the “accountability” and “personal responsibility” theme were as follows:

Stop screwing around and take ur [medication] now.

ChatGPT

It's impt to take care of urself. Pls take ur [medication]

Bard

ChatGPT and Bard did not reproduce 2 (29%) of the 7 human coders’ themes, “time” and “adherence,” which were defined rather literally as including the words “time” or “adherence” in the message. There were 4 instances where 2 themes derived by ChatGPT ultimately mapped onto a single broader theme identified by the human coders’ thematic analysis. For example, the human coders identified “community care,” which they defined as “message focused on the importance of the individual in relation to others, both being cared for by others and being accountable to others.” By examining both themes and their descriptions, we observed that there were 2 themes identified by ChatGPT that mapped onto “community care” as defined by our human coders:

1. *Supportive community*: messages highlighting the care and support from others
2. *Love and support from others*: messages emphasizing the impact on loved ones

### Reliability of Inductive Thematic Coding

The overall ICR of all inductive themes shared by human coding and ChatGPT was moderate (31/66, 47%). The overall ICR was lower between human coders and Bard at 37% (20/54), which is indicative of fair agreement. There was similarly fair agreement in coding between ChatGPT and Bard at 37% (23/62). There was notable variation in the ICR between coding arms when examined by theme, which ranged from 8% (2/26; slight agreement for “encouragement” between ChatGPT and Bard) to 80% (4/5; substantial agreement for “religious” between human coders and ChatGPT, human coders and Bard, and ChatGPT and Bard). Table 1 lists ICR between human coders, ChatGPT, and Bard for inductive thematic coding, both overall and by theme.

**Table 1:** Inductive thematic analysis intercoder reliability (ICR) between human coders, ChatGPT, and Bard by theme and overall.

Themes	Human coders and Bard			Human coders and ChatGPT			Bard and ChatGPT	
	Agreement, n/N (%; ICR)	Disagreement, n/N (%)	Agreement, n/N (%; ICR)		Disagreement, n/N (%)	Agreement, n/N (%; ICR)		Disagreement, n/N (%)
Encouragement	2/17 (12)	15/17 (88)	12/29 (41)		17/29 (59)	2/26 (8)		24/26 (92)
Health reminder	6/17 (35)	11/17 (65)	7/18 (39)		11/18 (61)	9/15 (60)		6/15 (40)
Religious	4/5 (80)	1/5 (20)	4/5 (80)		1/5 (20)	3/5 (60)		2/5 (40)
Community or cared by others	5/6 (83)	1/6 (17)	4/6 (67)		2/6 (33)	4/5 (80)		1/5 (20)
Warning	3/9 (33)	6/9 (67)	4/8 (50)		4/8 (50)	4/6 (67)		2/6 (33)
Personal responsibility (Bard and ChatGPT only)	—^a^	—	—		—	1/5 (20)		4/5 (80)
Overall (across all themes)	20/54 (37)	34/54 (63)	31/66 (47)		35/66 (53)	23/62 (37)		39/62 (63)

^a^Not applicable.

### Consistency of Deductive Thematic Analysis

A total of 12 themes were identified by the human coders following a deductive thematic analysis of the same text messages, which included “positive tone,” “stern/serious tone,” “sense of urgency/priority,” “balancing health with ‘fun’,” “self-care,” “expectations and attitudes,” “perceived negative outcomes” “perceived benefits,” “norms,” “social influence,” “self-efficacy,” and “spirituality/religion as motivation.” Of these 12 themes identified by human coders, 6 (50%) were also consistent with the themes derived by ChatGPT, and 7 (58%) were consistent with those found by Bard. Multimedia Appendix 2 lists the complete mapping of the deductive thematic analysis codebooks (including theme, description, and example SMS text messages) generated by human coders, ChatGPT, and Bard.

ChatGPT’s deductive thematic analysis identified a total of 9 themes, which included “consequences,” “health benefits,” “motivation,” “social influence,” “care and support,” “self-efficacy,” “religious beliefs,” “responsibility,” and “reminders.” Of these 9 themes, the majority (7/9, 78%) were consistent with the themes identified by our human coders. Bard’s inductive thematic analysis identified 6 themes from the SMS text messages, which included “importance of adherence,” “negative consequences of nonadherence,” “benefits of adherence,” “social support,” “self-efficacy,” and “religious/spiritual.” Of these 6 themes identified by Bard, there was perfect consistency (6/6, 100%) with the themes identified by human coders, and there was strong consistency (5/6, 83%) with the themes identified by ChatGPT.

ChatGPT and Bard did not reproduce 6 (50%) and 5 (42%), respectively, of the human coder’s 12 deductive themes. Neither ChatGPT nor Bard identified the human coder’s themes of “positive tone,” “stern/serious tone,” “balancing health with ‘fun’,” “self-care,” or “expectations and attitudes.” In addition, ChatGPT did not identify the human coder’s theme of “sense of urgency/priority,” which was defined as “includes messages instructing a person to place their health, or desired health behaviors, over other competing priorities.” On the basis of this description, the Bard theme of “importance of adherence” was mapped onto this theme, and both shared the example message of “Stop everything and take ur meds!”

There were 2 themes identified uniquely by ChatGPT (ie, neither Bard nor human coders identified these themes), which were “responsibility” and “reminders” and were defined as follows:

- Responsibility: encouraging a sense of responsibility for one’s health and well-being through adherence.
  - Example text: *It’s impt to take care of urself. Pls take ur [medication]*
- Reminders: providing reminders or cues to prompt medication adherence.
  - Example text: *Ready, set, get healthy! It’s med time. Time for ur [medication]*

There was 1 case where Bard identified a single theme (“social support”) that human coders and ChatGPT had separated into 2 separate themes (“norms” or “social influence” and “social influence” or “care and support,” respectively). Furthermore, there was an instance where 2 themes derived by ChatGPT ultimately mapped onto a broader theme identified by the thematic analysis performed by the human coders and Bard. For example, the human coders identified “perceived benefits,” which they defined as “perception of the effectiveness of an action to reduce the threat of illness or disease, including factors related to ease of use.” By examining both themes and their descriptions, there were 2 ChatGPT themes that mapped onto this theme of “perceived benefits”:

1. *Health benefits*: highlighting the positive impact of medication adherence on health and well-being
2. *Motivation*: encouraging individuals to take their medication by emphasizing the benefits of doing so

### Reliability of Deductive Thematic Coding

The overall ICR of deductive themes shared between human coders and ChatGPT was fair at 37% (22/59), which was similar to Bard at 36% (21/58). There was moderate agreement in coding between the codebooks generated by ChatGPT and Bard, as reflected by an overall ICR of 47% (22/47). We also examined code-specific ICR in addition to the overall ICR, which varied substantially across themes. For example, there was perfect (4/4, 100%) agreement in coding between human coders and ChatGPT, human coders and Bard, and ChatGPT and Bard within the theme of “perceived negative outcomes,” but only slight to fair agreement for the theme of “perceived benefits” (6/29, 21%; 5/13, 38%; and 9/28, 32%, respectively). Table 2 presents the ICR between human coders, ChatGPT, and Bard deductive thematic coding, both overall and by theme.

**Table 2:** Deductive thematic analysis intercoder reliability (ICR) between human coders, ChatGPT, and Bard by theme and overall.

Themes	Human coders and Bard			Human coders and ChatGPT			Bard and ChatGPT	
	Agreement, n/N (%; ICR)	Disagreement, n/N (%)	Agreement, n/N (%; ICR)		Disagreement, n/N (%)	Agreement, n/N (%; ICR)		Disagreement, n/N (%)
Perceived benefits	5/13 (38)	8/13 (62)	6/29 (21)		23/29 (79)	9/28 (32)		19/28 (68)
Perceived negative outcomes	4/4 (100)	0/4 (0)	4/4 (100)		0/4 (0)	4/4 (100)		0/4 (0)
Social Support (Norms and Social influence)	4/14 (29)	10/14 (71)	5/14 (36)		9/14 (64)	4/5 (80)		1/5 (20)
Self-efficacy	2/5 (40)	3/5 (60)	2/7 (29)		5/7 (71)	1/5 (20)		4/5 (80)
Spirituality or religion as motivation	4/5 (80)	1/5 (20)	5/5 (100)		0/5 (0)	4/5 (80)		1/5 (20)
Sense of urgency or priority (Bard only)	2/17 (12)	15/17 (88)	—^a^		—	—		—
Overall (across all themes)	21/58 (36)	37 (64)	22/59 (37)		37/59 (63)	22/47 (47)		25/47 (53)

^a^Not applicable.

### Total Time Spent on Qualitative Analyses

The human coding teams reported 492 (inductive) and 705 (deductive) total minutes to complete their thematic analyses of the SMS text messages. This total time includes the sum of the time spent by each individual coder on their initial coding, codebook development, application of codebook, and reaching consensus on disagreements in coding. The total time to complete the inductive and deductive thematic analyses with ChatGPT was 15 minutes (97% less time than the human approach) and 25 minutes (97% less), respectively, whereas both analyses took a total of 20 minutes with Bard (96% and 97% less time, respectively). The total time spent on thematic analyses using GenAI was the sum of the time taken to input the prompts, wait for responses to generate, and document those responses in a spreadsheet table.

## Discussion

### Principal Findings

This study evaluated the consistency and ICR in themes between human coders and GenAI models conducting both inductive and deductive thematic analyses of short SMS text message prompts that were used in a previous intervention to promote medication adherence. There was evidence of consistency in the themes identified by ChatGPT and Bard compared to human coders’ inductive thematic analysis (both 5/7, 71%), but the consistency was notably lower for deductive thematic analysis (6/12, 50% and 7/12, 58%, respectively). The overall ICR (percent agreement in coding) of themes shared between human coders and GenAI models (inductive: 31/66, 47% and 20/54, 37%; deductive: 22/59, 37% and 21/58, 36%, respectively) was fair to moderate [31]. In addition, GenAI models were significantly less resource-intensive, as they took an average of 97% less time (20 vs 567 min) for qualitative analysis compared to human coders. ChatGPT and Bard performed similarly to each other across both types of thematic analysis.

This study is the first of our knowledge to compare the GenAI- and human-generated themes from textual data following both inductive and deductive qualitative thematic analysis procedures using health-related data. We also evaluated and compared both ChatGPT and Bard, whereas prior studies of GenAI have only examined ChatGPT. Our findings demonstrate that GenAI may provide a promising opportunity to facilitate quicker and more resource-efficient qualitative analysis of textual data; however, such technologies should be used to assist human coders in order to further improve the efficacy and reliability of findings.

### Comparison With Prior Work

Although we did not find perfect consistency in AI- and human-generated themes, there were notable similarities in the themes derived by both methods. Hamilton et al [20] similarly compared emergent ChatGPT- and human-generated themes from a qualitative analysis of interview data from a guaranteed income program evaluation, in which they also found an overlap between the 2 methods. They found that approximately 50% of human-generated themes were consistent with those identified by ChatGPT and that 80% of themes identified by ChatGPT were identified by human coders. Furthermore, de Paoli [11] emulated inductive thematic analysis of a previously analyzed semistructured interview data set using the underlying natural language processing (NLP) model of ChatGPT (GPT 3.5-Turbo) and found that a majority of the original themes (9/13, 69%) were identified. The consistency between our GenAI- (ChatGPT and Bard) and human-generated thematic analyses (50%-71%) was notably similar to that observed in these studies (50%-80%) [11,20]. The results of this study and the study by Hamilton et al [20] both found that both GenAI- and human-generated themes were promisingly similar, but both methods also identified distinct themes. Although de Paoli [11] and Hamilton et al [20] had previously demonstrated and evaluated the potential efficacy of GenAI for qualitative research, our findings further suggest that GenAI may also have promising applications for qualitative research in the context of health research.

In this study, the data set consisted of SMS text messages to promote HIV medication adherence for individuals who use methamphetamine and included nuanced references that are unique to this population such as references to substance use (eg, “fun” and “partying”) and specific slang for methamphetamine (“Tina”). The deductive human coding team identified the theme of “balancing health with ‘fun’” based on these messages and recognized the nuance of the use word “fun” in this context as a subtle reference to substance use (codebook description: “Messages contain content reminding a person to prioritize health, even engaging in ‘fun’ or ‘partying behaviors,’ which may include risky behaviors”), whereas ChatGPT and Bard did not. For these messages, both AI methods tended to take a literal meaning and labeled these messages as representing themes of “fun and enjoyment” or “enjoyment.” However, it is also important to recognize that our inductive coding team similarly did not appear to recognize these subtle references to substance use behaviors. In terms of other notable discrepancies in themes, the human-generated deductive themes included “positive tone” and “stern/serious tone,” which neither ChatGPT nor Bard produced. These themes appear to be consistent with sentiment analysis (or recognizing the sentiment or emotion expressed in text), which is surprising given that recent research has found ChatGPT to be quite promising in sentiment classification of textual data (>92% accuracy) and superior to other NLP methods [32].

One possible explanation for the difference in the results between methods is that GenAI methods appear to be relatively limited in their ability to understand the contextual or subtle meaning of textual data, as they rely primarily on probabilistic pattern recognition to generate responses. The training sets used to train the NLP models underlying ChatGPT and Bard (ie, largely internet content) presumably did not contain a substantial amount of textual data specific to substance use, and so the ability to recognize subtle nuances and references within this context is more limited. The implications of these findings suggest that GenAI shows promise in qualitative thematic analysis but may ultimately prove less valid for less mainstream research topics that may relatively use more nuanced language (eg, illicit substance use), which further highlights the importance of continued inclusion of human coders in the qualitative research process. This possible limitation regarding the more explicit interpretation of GenAI that we observed appears to extend to not only the output it produces but also its use of input (prompts). Whereas our human coders’ understanding of qualitative thematic analysis included the possibility of themes emerging related to the sentiment of the text messages, it appears that ChatGPT and Bard did not. This finding highlights the relative importance of prompt engineering (ie, research into how best to instruct such technologies) and further stresses the importance of maintaining human coders in the qualitative research process when leveraging GenAI.

Currently, there is considerable heterogeneity in the prompts being used to conduct qualitative research with GenAI to date, such that some examples have had the models show their work and used step-by-step prompts following a typical 6-step process [11], and others have been more global in their approach [20] similar to ours. While general guidance exists on how best to prompt GenAI in the context of health care [29], we believe that this represents a critical future direction of research for this field of work. As more studies and case examples are published, a systematic review would be critical for developing best practices and standards for prompt engineering in the specific context of qualitative analysis (eg, are step-by-step prompts better received than more global ones such as the ones used in our study?).

Given that GenAI and human coders were operating off independently derived codebooks and themes, the degree of disagreement in ICR is not surprising. After the initial application of their codebooks and before meeting and coming to a consensus on disagreements, our inductive and deductive human coding teams had ICRs of 83% (57/69) and 31% (45/144), respectively. Therefore, the ICRs found between our human coders before discussing disagreements was in fact quite similar to the ICRs we observed between human and GenAI methods for shared themes. Furthermore, Xiao et al [33] similarly examined the degree of ICR between a pretrained LLM (GPT-3) and deductive coding conducted by expert human coders, in which they also found fair to substantial agreement between methods [33]. Regarding the difference in ICRs between the inductive and deductive analyses, current literature suggests that this most likely is a reflection of the method and the associated number of codes. In our study, the human deductive analysis team identified more themes (and codes) than did the inductive analysis team (12 vs 7, respectively), which could be suspected as they had been provided with a priori codes from several theories of behavior change. Previous research has found that a greater number of codes reduces the ICR [34,35], which is believed to reflect having to be familiar with a relatively longer coding scheme and thus being more cognitively taxing [36]. There is also substantial debate over the utility of ICR in qualitative methods, as some argue that the inherent subjectivity of qualitative research and the resulting researcher’s reflexivity and personal engagement are necessary for understanding the diversity of perspectives on a given topic rather than treating it as noise to be minimized [36,37]. The arguments in support of using ICR are that it helps ensure that themes and information being derived from qualitative data are consistent and meaningful [38]. Therefore, GenAI-generated qualitative analyses may be useful as tools for providing an additional perspective of the data to complement those found by human coders and enabling triangulation and recognition of potential biases.

A significant barrier to qualitative analysis is the considerable time and resources involved, which can be particularly salient when rapid research findings are urgently needed to improve the dissemination and implementation of evidence-based health interventions [4,39]. Previous innovations in qualitative methods, such as rapid qualitative analysis, have shown promise in helping maintain the rigor of the analysis while being quicker and more cost-efficient than traditional methods [1,7]. However, such methodologies still require substantial human resources, and the resource efficiency of qualitative methodologies may be further improved when augmented with new technologies. Several examples of hybrid NLP-qualitative methods, whereby human coders and NLP or GenAI technologies collaborate during analysis, have been proposed or demonstrated previously [40-43]. Skeen et al [41] have provided one such example of a hybrid approach in their proof-of-concept study that applied NLP to condense a large data set of unstructured textual data before subsequent human-generated thematic analysis in order to more rapidly produce design insights for improving a digital HIV intervention.

However, most of these studies using hybrid methods have only demonstrated proof of concept and lacked comparisons with gold-standard qualitative analysis conducted by human analysts. In one previous study comparing qualitative analysis with human coders, NLP-only, and NLP-hybrid methods, the authors found similar thematic findings across methods and that NLP and hybrid methods required notably less time and resources [43]. Whereas the technical skills (eg, coding) required to implement NLP methods previously posed a significant barrier to the wider adoption of such methodologies among qualitative researchers, commercially available GenAI services, such as ChatGPT and Bard, provide a promising opportunity for further exploration of hybrid NLP-qualitative methods. An example of a hybrid approach incorporating GenAI might be for it to complete the often time-intensive initial coding of textual data, which could subsequently be reviewed and summarized by human analysts to produce the themes that are often more interpretative and abstract in nature. Alternatively, a single human coder might conduct a complete thematic analysis and then collaborate with GenAI as if they were another human coder to reflect on discrepancies and convergence between their coding and identification of broader themes (ie, replacing the need for a second human coder or analyst). The unknown feasibility, efficacy, and efficiency of such hybrid approaches leveraging GenAI warrant future exploration and study.

### Limitations

There are several important limitations to consider when interpreting the findings of our study and more broadly the application of GenAI to qualitative analysis. First and foremost, there are numerous current ethical and privacy issues to applying GenAI to human participant research. These issues are currently being debated as these technologies emerge and include the potential for perpetuating bias and inequality, fact fabrication, plagiarism, and potential breaches of data privacy or ownership [44-48]. Using GenAI in the research process also poses potential challenges to obtaining informed consent from participants, especially when working with at-risk populations such as those living with HIV or those who use substances. Obtaining informed consent is fundamental to the ethical conduct of research and involves disclosing to potential participants how their data could be used and the risks associated with research participation, both of which may be difficult to do in the context of using GenAI services due to their lack of transparency, explainability (eg, black box), and the potential risk of reidentification [49]. Future researchers should continue to prudently investigate and monitor both the potential benefits and risks associated with groundbreaking technologies such as GenAI services, especially when incorporating them into the scientific process and their use with data from vulnerable populations.

In addition, it is important to note that our data set consisted of only relatively brief SMS text message prompts that could easily be provided to ChatGPT and Bard. We do not know how well the consistency and reliability of themes derived by GenAI would compare to human coders for longer textual data sets that are common in qualitative research (ie, unstructured or structured interview, focus group transcripts, etc). Our data set also notably did not consist of natural, participant-generated language (eg, transcribed spoken language in interviews), so our findings may not generalize to these more likely data sets for qualitative analysis. However, recent studies have conducted qualitative analyses using GenAI services or their underlying LLMs with such data sets (eg, unstructured qualitative interview transcripts and significant statements from transcripts) and have shown promisingly similar results to ours [11,20].

Relatedly, we observed challenges with GenAI being able to recognize interpretative themes and consider the nuanced meaning of some topics (specifically, substance use), which may also suggest that our findings may not generalize to all research content areas. Although the flexibility of thematic analysis allows and expects to some degree that initial codes go on to form main themes [2,50], our study was relatively limited in the extent to which we could determine whether GenAI correctly identifies more complex themes due to our small data set of brief text messages. Given the limited research that exists examining the consistency and reliability of applying GenAI to qualitative research and the novelty of the field, future studies should consider further exploring how well these methods generalize to other types and content of data.

### Conclusions

Our findings suggest that GenAI may have promising applications for qualitative thematic analysis (including reducing the time and resources required), but hybrid approaches that allow for collaboration between human coders and GenAI technologies are likely necessary to further improve the consistency and reliability of such methods. Improvements in efficiency may be particularly important to further facilitating the adoption of qualitative methods for studying and improving digital health interventions within often complex and rapidly changing real-world settings. As GenAI models are expected to continually improve as they learn, future studies should further explore how humans can best collaborate with these powerful tools given their potential for enabling more rapid research while also remaining vigilant of the potential risks they may pose. Research into the ethical challenges posed by GenAI in the context of human participant research is also urgently needed.

## Appendix

Multimedia Appendix 1 Inductive thematic analysis codebooks generated by human coders, ChatGPT, and Bard.

Multimedia Appendix 2 Deductive thematic analysis codebooks generated by human coders, ChatGPT, and Bard.

## Abbreviations

AI: artificial intelligence
GenAI: generative artificial intelligence
ICR: intercoder reliability
iTAB: individualized texting for adherence building
LLM: large language model
NLP: natural language processing
