# Supplementary material for: Comparing the Efficacy and Efficiency of Human and Generative AI: Qualitative Thematic Analyses
Source: JMIR AI. 2024 Aug 2;3:e54482. doi: 10.2196/54482 (PMC11329846; doi:10.2196/54482)
Supplement: Multimedia Appendix 1 [file ai_v3i1e54482_app1.docx]

| Multimedia Appendix 1: Human, ChatGPT, and Bard-generated Inductive Thematic Analysis Codebooks | | | | | | | | |
| --- | --- | --- | --- | --- | --- | --- | --- | --- |
| **Human** | | | **ChatGPT** | | | **Bard** | | |
| *Theme* | *Description* | *Example* | *Theme* | *Description* | *Example* | *Theme* | *Description* | *Example* |
| Time | Message explicitly uses the word "time" to indicate the moment when a person should take their meds | It's med time! Pls take ur [medication]; It's pill time! Take ur [medication] |  |  |  |  |  |  |
|  |  |  |  |  |  |  |  |  |
| Adherence | Message explicitly mentions adherence | Adherence is impt. Pls take ur [medication] |  |  |  |  |  |  |
| Religious | Message revolves around faith and includes words such as god, lord, jesus, etc. | The Lord believes in u; believe in urself. Take ur [medication] | Spirituality/Higher power | Messages incorporating religious or spiritual beliefs. | Trust in the Lord. Take ur [medication] | Religious/spiritual beliefs | The messages appeal to people's religious or spiritual beliefs by suggesting that God loves them and wants them to be healthy. They also suggest that people should trust in God and believe in themselves when they are making decisions about their health. | God loves u. It's time 4 ur [medication]  Trust in the Lord. Take ur [medication]  The Lord believes in u; believe in urself. Take ur [medication] |
| Community care | Message focuses on the importance of individual in relation to others, both being cared for by others and being accountable to others | People care about u. Pls take ur [medication] | Supportive community | Messages highlighting the care and support from others. | We care about u and ur health. Pls take ur [medication] | Social support | The messages remind people that others care about them and want them to be healthy. They also suggest that people think about the people who love them when they are deciding whether or not to take their medication. | People care about u. Pls take ur [medication]  Think about the ppl who love u. Take ur [medication] |
|  |  |  | Love and support from others | Messages emphasizing the impact on loved ones. | Think about the ppl who love u. Take ur [medication] |  |  |  |
| Health Reminder | Message includes the word "health" and promotes awareness of an individual's health, longevity, and self-care/efficacy | 2 control ur health u need 2 take ur [medication] | Health benefits | Messages emphasizing the positive impact on health and well-being. | 2 control ur health u need 2 take ur [medication] | Importance of taking medication | The messages emphasize the importance of taking medication to stay healthy, feel good, and live longer. | Stay healthy! It's time 2 take ur meds, pls take ur [medication]  Live longer! Take ur [medication] |
|  |  |  | Reminder | Reminders emphasizing the importance of taking medication. | Yay, health! Don't frgt 2 take ur [medication |  |  |  |
| Warning | Message that includes a negative or serious tone, alerts the individual of health consequences, may include words such as "stop," "don't," "not," or "now" | Not taking ur meds could make u resistant. Take ur [medication] | Resistance and risk to others | Messages highlighting the risks of non-adherence for others. | Not taking ur meds puts others at risk. Pls take ur [medication] | Consequences of not taking medication | The messages warn people about the serious consequences of not taking medication, such as getting sicker, becoming resistant to medication, and putting others at risk. | Not taking ur meds has severe consequences. Take ur [medication]  Not taking ur meds could make u resistant. Take ur [medication] |
|  |  |  | Consequences of non-adherence | Messages warning about the negative consequences of not taking medication. | Not taking ur meds has severe consequences. Take ur [medication] |  |  |  |
| Encouragement | Message of reassurance, encouragement, praise, and may include words such as fun, party, or yay. Also may include an exclamation point. | U are special. Pls take ur [medication] | Positive reinforcement | Encouraging and positive messages to motivate medication adherence. | U are special. Pls take ur [medication] | Enjoyment | The messages suggest that people can still enjoy themselves and have fun even if they are taking medication. They also suggest that taking medication is a way to protect oneself from the negative consequences of not taking medication. | Take ur meds so u can keep having fun. Time 4 ur [medication]  You can have fun and take ur meds. Time 4 ur [medication] |
|  |  |  | Fun and enjoyment | Messages encouraging the notion that taking medication can still be enjoyable. | You can have fun and take ur meds. Time 4 ur [medication] |  |  |  |
|  |  |  | Accountability | Messages emphasizing personal responsibility for adherence. | Stop screwing around and take ur [medication] now. | Personal responsibility | The messages emphasize that it is important for people to take care of themselves and take their medication on their own. They also suggest that people should be proud of themselves for being adherent to their medication regimen. | It's impt to take care of urself. Pls take ur [medication]  U've been doing gr8 w/ ur adherence. It's time 2 take ur [medication] |
