# Supplementary material for: Comparing the Efficacy and Efficiency of Human and Generative AI: Qualitative Thematic Analyses
Source: JMIR AI. 2024 Aug 2;3:e54482. doi: 10.2196/54482 (PMC11329846; doi:10.2196/54482)
Supplement: Multimedia Appendix 2 [file ai_v3i1e54482_app2.docx]

| Multimedia Appendix 2: Human, ChatGPT, and Bard-generated Deductive Thematic Analysis Codebooks | | | | | | | | |
| --- | --- | --- | --- | --- | --- | --- | --- | --- |
| **Human** | | | **ChatGPT** | | | **Bard** | | |
| *Theme* | *Description* | *Example* | *Theme* | *Description* | *Example* | *Theme* | *Description* | *Example* |
| Positive tone | Tone of message evokes positive feelings like pride, inspiration, hope, optimism, etc. Messages contain content suggesting that life factors, including health, will be satisfactory in the future- especially as a result to a desired health behavior. Messages can also be optimistic in their tone- such as messages that aim to contextualize health behaviors as proactive, positive efforts. | U are special. Pls take ur [medication] |  |  |  |  |  |  |
| Stern / serious tone | Messages are written in an encouraging but austere manner. | Stop screwing around and take ur [medication] now. |  |  |  |  |  |  |
| Sense of urgency / priority | includes messages instructing a person to place their health, or desired health behaviors, over other competing priorities. | Stop everything and take ur meds! Take ur [medication] |  |  |  | Importance of adherence | Messages that stress the importance of taking medication, regardless of other factors. | No matter what else is going on, it's impt 2 take ur [medication]  Stop everything and take ur meds!  It's impt to take care of urself. Pls take ur [medication] |
| Balancing health with "fun" | Messages contain content reminding a person to prioritize health, even engaging in "fun" or "partying behaviors", which may include risky behaviors. | Take ur meds so u can keep having fun. Time 4 ur [medication] |  |  |  |  |  |  |
| Self-care | Mentions specific health behaviors as a form of "self-care", to promote general wellbeing. | It's impt to take care of urself. Pls take ur [medication] |  |  |  |  |  |  |
| Expectations and attitudes | This refers to the anticipated consequences and outcomes of a performing a behavior of interest. Expectations and attitudes to outcomes may be health related or may address other aspects of a person's wellbeing. | Live longer! Take ur [medication] |  |  |  |  |  |  |
| Perceived negative outcomes | Perception of risk of contracting an illness or disease, or leaving an illness or disease untreated May include medical consequences (e.g., death, disability) and social consequences (e.g., family life, social relationships) when evaluating the severity. | U will only get sicker if u don't take ur meds. Time for ur [medication] | Consequences | Highlighting the potential negative outcomes of not adhering to medication. | Not taking ur meds has severe consequences. Take ur [medication] | Negative consequences of non-adherence | Messages that warn about the negative consequences of not taking medication, such as getting sicker, becoming resistant to medication, and putting others at risk. | Not taking ur meds has severe consequences. Take ur [medication]  Not taking ur meds could make u resistant. Take ur [medication]  U will only get sicker if u don't take ur meds. Time for ur [medication] |
| Perceived benefits | Perception of the effectiveness of an action to reduce the threat of illness or disease, including factors related to ease of use. | It only takes a second! Take ur [medication] | Health benefits | Highlighting the positive impact of medication adherence on health and well-being. | Stay healthy! It's time 2 take ur meds, pls take ur [medication] | Benefits of adherence | Messages that emphasize the positive consequences of taking medication, such as feeling good, living longer, and protecting one's health. | Stay healthy! It's time 2 take ur meds, pls take ur [medication]  Live longer! Take ur [medication]  Ur health is impt, rmber 2 take ur meds. Take ur [medication] |
|  |  |  | Motivation | Encouraging individuals to take their medication by emphasizing the benefits of doing so. | U can do it! Take ur [medication] |  |  |  |
| Norms | Refers to the customary codes of behavior or values held in a group of people or in a larger cultural or societal context. Norms are considered normative, or standard, in a group of people. | Ur health is impt, rmber 2 take ur meds. Take ur [medication] | Social influence | Using social norms and relationships to influence adherence behavior. | Think about the ppl who love u. Take ur [medication] | Social support | Messages that highlight the importance of social support in promoting adherence, such as the support of family and friends. | People care about u. Pls take ur [medication]  Think about the ppl who love u. Take ur [medication]  We care about u and ur health. Pls take ur [medication] |
| Social influence | Process where people directly or indirectly influence thoughts, feelings and action of others. This may include the influence of important persons such as close relatives, or friends. | [name] needs you. Take ur [medication] for them. | Care and support | Conveying care, concern, and support from others to motivate adherence. | People care about u. Pls take ur [medication] |  |  |  |
| Self-efficacy | Refers to a person's confidence and real-world ability to successfully perform a behavior. Includes a person's perceived difficulty of the behavior, and their perceived knowledge / mastery of how to do the behavior efficaciously. | U've been doing gr8 w/ ur adherence. It's time 2 take ur [medication] | Self-efficacy | Building confidence in one's ability to adhere to the medication regimen. | It only takes a second! Take ur [medication] | Self-efficacy | Messages that emphasize the individual's ability to take medication, such as their ability to overcome challenges and stay on track. | U can do it! Take ur [medication]  It's a great accomplishment to be adherent! Take ur [medication] |
| Spirituality / religion as motivation | Mentions religion, religious figures, and spirituality to help encourage a person to perform a desired health behavior. | Trust in the Lord. Take ur [medication] | Religious beliefs | Incorporating religious beliefs or spirituality to encourage adherence. | God grant me the serenity to do this. It's time 4 ur [medication] | Religious/spiritual | Messages that appeal to religious or spiritual beliefs, such as the belief that God wants people to take their medication. | God loves u. It's time for ur [medication]  WWJD? Take his meds! Time 4 ur [medication]  Trust in the Lord. Take ur [medication]  The Lord believes in u; believe in urself. Take ur [medication] |
|  |  |  | Responsibility | Encouraging a sense of responsibility for one's health and well-being through adherence. | It's impt to take care of urself. Pls take ur [medication] |  |  |  |
|  |  |  | Reminders | Providing reminders or cues to prompt medication adherence. | Ready, set, get healthy! It's med time. Time for ur [medication] |  |  |  |
